# Hemodynamic responses of the prefrontal cortex following cold water immersion during an eyes-closed balance assessment

**DOI:** 10.1007/s00421-025-06027-2

**Published:** 2025-10-23

**Authors:** Cory Smith, Owen Salmon, Matt Segovia, Thomas Statz, Cierra Ugale, Rachel Rauth, Michael Mastrodicasa, Jaeho Shim

**Affiliations:** Baylor University, Waco, TX USA

**Keywords:** CWI, fNIRS, Postural stability, Thermal strain

## Abstract

**Purpose:**

This study aimed to examine the impact of cold water immersion (CWI) on balance and the corresponding hemodynamic responses that occur within the prefrontal cortex (PFC). Measuring PFC activation during balance will identify cognitive mechanisms underlying postural control, which can be used to identify balance impairments or fall risk.

**Methods:**

Twenty-three participants completed two separate testing visits consisting of a 10-min CWI at 15 °C and thermoneutral water immersion (TWI) at 35 °C. A 30-s eyes-closed balance assessment was performed pre- and post-exposure to identify changes in Path Length, Sway Range, and Sway Velocity. Oxygenated (O_2_Hb) and deoxygenated hemoglobin (HHb) were measured over the PFC using functional near-infrared spectroscopy during all balance assessments.

**Results:**

There were no detectable differences for Sex in any variable, nor significant differences in Path Length (*p* = 0.94), Sway Range (*p* = 0.92), and Sway Velocity (*p* = 0.81) between the CWI and TWI exposures. For the TWI, there were no differences in O_2_Hb (*p* = 0.15) or HHb (*p* = 0.28) between the pre- and post-exposure balance assessments. The CWI exposure resulted in a significant decrease in O_2_Hb (*p* ≤ 0.01) and an increase in HHb (*p* ≤ 0.01).

**Conclusion:**

The demands of thermoregulation and balance maintenance following CWI resulted in distinct cerebral hemodynamic patterns which potentially reflected increased neural demand during the tasks. Increase in HHb and decrease in O_2_Hb suggest a unique response during the refractory period between the CWI and balance assessment that may reflect competing thermoregulatory processes associated with rewarming.

## Introduction

Cold environments present unique physiological and operational challenges which have been linked to increased risk of musculoskeletal injuries (MSKI) ; Farbu et al. 2022). For example, Patterson 2018 reported that emergency department visits increased 2–5% across all ages during colder winters compared to warmer winters in the United Kingdom. In addition, Dybin et al. 2022 indicated that MSKIs were the second leading cause of death (13%) associated with cold-weather injuries in the Arctic, following respiratory complications (50%). The increased risk of MSKI due to cold exposures may be due to impaired or a dysregulation of the brain, which would reduce the ability to perform the motor tasks required to maintain balance (Oksa et al. 1997; Rutkove 2001; Castellani and Young 2016; Scott et al. 2016; Short 2019). While these studies emphasize the significant impairment of peripheral neuromuscular systems due to cold exposure, there is limited research available on how cold exposure negatively affects the functional capacity of the brain. Specifically, cold exposure can result in neural dysregulation in the brain during memory tasks (Palinkas 2001; Falla et al. 2021), cognition, attention (Falla et al. 2021; Jones et al. 2022), and processing tasks (Palinkas 2001). The combination of peripheral and central nervous system impairments all contributes to the increased MSKI risk during cold exposures.

Impaired peripheral and central nervous systems are of particular importance to Service Members (SM), miners, and maritime workers in the Arctic and sub-Arctic regions where the risk of cold water exposure is high and is coupled with limited medical transport, supplies, or provider support when injuries occur (McKenzie et al. 2024). Cold water immersion (CWI) can be more dangerous than cold air exposure as water cools the body up to 25 times faster than that of air exposure, making CWI an optimal approach to examine the effects of cold exposure on performance and injuries (O’Brien et al. 2000; Castellani and Young 2016; Deshmukh and Lilariya 2019). The ability to maintain postural control and balance in extreme cold-weather conditions is crucial for occupational and military performance, but the majority of research has focused on peripheral physiological responses or cognitive-related processing (Falla et al. 2021; Jones et al. 2022). Understanding how CWI affects balance and neural activation will help us to identify the underlying mechanisms impairing performance and aid in the development of targeted countermeasures to maintain military readiness and safety in cold-weather operations (Ojanen et al. 2024).

Functional near-infrared spectroscopy (fNIRS) is a non-invasive neuroimaging tool that measures the hemodynamic responses associated with the metabolic demand of the brain (Pierro et al. 2014; Bonilauri et al. 2021). The use of fNIRS allows for reliable measurements of neural activation during cold exposure studies and during dynamic movement assessments (Kalia et al. 2018; Han et al. 2023; Sharooni et al. 2024). For example, Kalia et al. 2018 reported greater fNIRS-derived oxygenated hemoglobin (O_2_Hb) amplitude fluctuations over the prefrontal cortex (PFC) in males following a 3-min CWI at 1–3 °C compared to a thermoneutral water immersion (TWI) when performing the Wisconsin Card Sorting Test. Similarly, Sharooni et al. 2024 identified a significant correlation using fNIRS between O_2_Hb and cognitive task performance over the prefrontal cortex during a 30-min cold air exposure (19 °C); however, the authors did not specify whether this correlation was positive or negative, and no detectable changes were observed during the resting state or thermoneutral condition. These studies demonstrated the utility of fNIRS in cold exposure physiological monitoring; however, greater exploration of the real-time hemodynamic and neurophysiological responses is needed to develop strategies to improve performance. Therefore, this study aimed to examine the impact of CWI on balance and the corresponding hemodynamic responses that occur within the PFC as measuring PFC activation during standing balance provides valuable insights into cortical involvement in postural control (Kohli et al. 2025). We hypothesized that CWI would impair balance performance (i.e., greater path length, sway range, and sway velocity) and increase PFC activation (i.e., greater HHb, reduced O_2_Hb) compared to TWI.

## Methods

### Participants

Twenty-six participants (13 females, 13 males) were recruited for this study and completed all testing visits, with their demographics described in Table 1. There were no dropouts, and all 26 participants completed both testing visits. All participants were free of any known pulmonary diseases, musculoskeletal injuries, cardiovascular diseases, or seizure disorders. In addition, all participants were naive to cold exposure therapies, including CWI, environmental chambers, or recent travel to cold climates. Participants completed informed consent and underwent a structured familiarization session prior to their testing visits, during which they were instructed on proper balance positioning and completed one to two practice trials to ensure task understanding. The order of experimental visits was randomized and counterbalanced so that some participants completed the TWI visit first and others the CWI first. This study was approved by the university’s institutional review board (IRB: 2,115,890) and in accordance with the Declaration of Helsinki (WMA 2013).

**Table 1:** Descriptive statistics (Mean ± SD) for the participants age, height, weight, and body mass index (BMI)

Measure	Males	Females
*N*	13	13
Age (yrs)	24.1 ± 4.1	23.2 ± 4.1
Height (cm)	180.2 ± 6.3	166.3 ± 5.4
Weight (kg)	82.3 ± 10.6	66.9 ± 9.3
BMI (kg/m^2^)	25.3 ± 2.2	24.2 ± 3.0

### Experimental design

This study investigated the effects of short-term, whole-body, head-out, CWI on balance performance and PFC hemodynamic-derived neural activation. Twenty-six participants, equally distributed between males and females, underwent two randomized water immersions consisting of a TWI (35 °C) and CWI (15 °C) exposure condition (Ntoumani et al. 2023). The TWI and CWI conditions were performed within seven days of each other and at approximately the same time of day (1.20 ± 1.34 h) to account for diurnal thermogenetic variations (Straat et al. 2022). For each condition, participants wore the same clothing, consisting of swim trunks for males and a combination of a sports bra and spandex shorts for females. To assess PFC activation, each participant was fitted with a fNIRS device. Prior to the immersion protocol, a baseline balance assessment was conducted using a pressure mat, where participants performed a 30-s balance test standing in a heel-to-toe staggered stance, barefoot, with their hands at their sides and eyes closed. Upon completion of the balance assessment, the participants transitioned into a thermally controlled tub where they submerged their whole body into the water until level with the clavicles for a total duration of 10 min. Upon completion of the 10-min immersion, the participants exited the water, quickly dried off, and returned to the pressure mat to complete a secondary 30-s balance assessment (Silva et al. 2012; de Abreu et al. 2024). The 30-s balance assessment was utilized as it showed the ability to identify the risk of injury and performance while minimizing the confounding effects of thermal recovery on neuromuscular function and peripheral vasodilation (Brazaitis et al. 2016; Tan and Knight 2018; Nazari et al. 2020).

### Cold exposure

An inflatable cold plunge tub with a recirculating chiller was used for all CWI visits to accurately maintain the thermoneutral (35 °C) and cold conditions (15 °C) (Revive Cold Plunge, United States). A CWI temperature of 15 °C for 10 minutes was utilized based on previous research that has shown it to induce a cold shock response and allow for the examination of the interplay between PFC hemodynamic responses and balance performance (Vieira et al. 2016; Wang et al. 2025). For both conditions, the circulating pump continuously ran to circulate the water throughout the tank. The inlet was located at the bottom left and the outlet was located at the top right of the tank. This allowed for continuous circulation and stabilization of the water temperature throughout to maintain the target temperatures. During each water immersion session, participants were required to sit in the tank with their clavicles at water level for a duration of 10 minutes. The timer started as soon as participants were seated at the correct water depth. They were instructed not to let any part of their body exit the water, avoid creating friction for warmth, and refrain from adopting positions, such as the fetal position, that could help maintain body temperature.

### Balance

Balance was assessed Pre- and Post-Exposure during the TWI and CWI visits. Path Length, Sway Range, and Sway Velocity were calculated during a 30-s period where participants staggered their feet in a straight line (toe to heel) on the 43.2 cm × 36.8 cm MatScan pressure mat (Tekscan Inc., Norwood, MA, United States; sampling rate = 100 Hz) with their eyes closed until the completion of the test. Path Length was calculated as the cumulative distance of the center of pressure (COP) during the 30-s measurement period. Sway Range was calculated as the maximum excursion of the COP in both the anterior–posterior and medio-lateral directions using 95% confidence interval ellipses around the COP. Anterior–Posterior Sway Index was measured as the variation in the COP using the Root Mean Square Error calculated from the distance of each COP from the mean position.

### Functional Near-Infrared Spectroscopy (fNIRS)

**Fig. 1:**
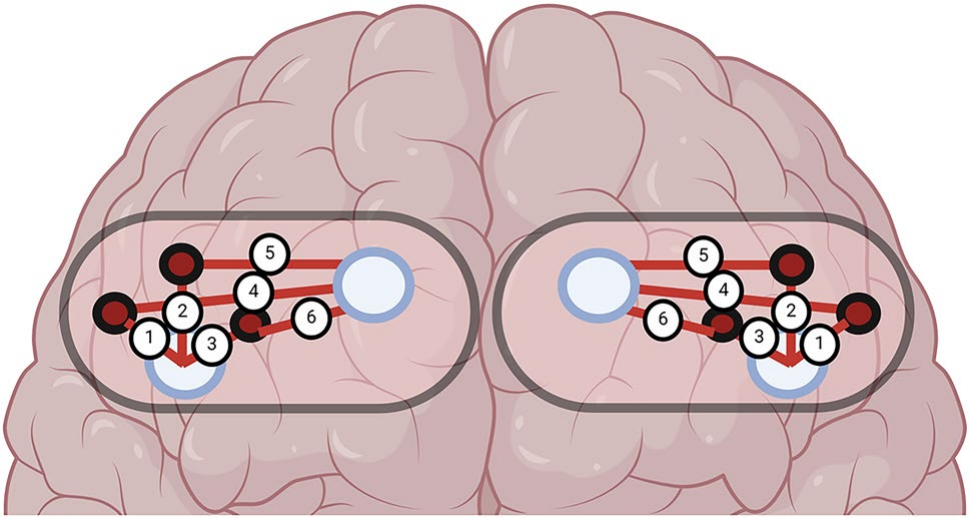
The figure depicts the layout of the long and short channels. The large blue circles represent the detectors, the small red and black indicate the sources and the red numbered lines show their path of data collection

During the balance task, PFC activation was measured using two continuous-wave fNIRS sensors (PortaLite MKII, Artinis Medical Systems B.V., Elst, the Netherlands) bilaterally attached to the forehead approximately 2 cm above the eyebrows. The sensors were secured to the skin using double-sided adhesive tape and then wrapped by a black elastic pre-wrap bandage to further secure the sensors to the forehead and protect the sensors from ambient light contamination. The placement of the sensors was similar to previous studies (Yennu et al. 2016; Klop et al. 2023) corresponding to the two frontopolar (Fp1 and Fp2) regions of the PFC based on the international 10–20 system (Acharya et al. 2016). Each sensor consisted of three light-emitting diodes, which emit light at two wavelengths (760 nm and 850 nm), and two detectors placed at inter-optode distances of 29, 35, and 41 mm for the three long channels and 7, 7.4, and 8 mm for the three short channels. The control unit was placed in a fanny pack secured around the participant’s waist during the balance task (Fig. 1).

Relative concentration changes in fNIRS-derived cerebral hemodynamics (O_2_Hb and HHb) were acquired in Oxysoft (version 3.4, Artinis Medical Systems B.V., Elst, the Netherlands) at a sampling frequency of 50 Hz. The relative concentrations were computed from the original optical densities using the modified Beer–Lambert law equation, adjusted for the age-dependent differential path-length factor, calculated for each participant (4.99 + 0.067*(Age^0.814^)). Within the Oxysoft software, each signal was low-pass filtered at 0.14 Hz to remove physiological noise components (e.g., heart rate, respiration, Mayer waves). The selection of this filter was based on a visual inspection of the power density spectrum of the data and previous fNIRS recommendations (Klein and Kranczioch 2019; Patashov et al. 2023). After filtering the signals, the relative concentration data were exported and further analyzed in LabVIEW (version 2023, National Instruments, Austin, TX). Within the LabVIEW program, channels with low signal quality were removed, and short-separation channels were regressed from the long channels (Sato et al. 2016). Additionally, the average O_2_Hb and HHb concentrations obtained during the 5-s eyes-open pre-task period were used as baseline values. The 5-s window was selected to avoid unnecessarily elongating the 30-s balance task and is consistent with a prior fNIRS study examining standing balance (Kohli et al. 2025). These baseline values were subtracted from the average concentrations obtained throughout the 30-s balance task to assess relative change in O_2_Hb and HHb during each trial. Because there were no significant differences between the right and left PFC regions, the regions from both sides were averaged together to represent overall PFC activation.

### Statistical analysis

Two separate multivariate analysis of variance (MANOVA) were performed for the Balance and fNIRS assessments. One MANOVA assessed balance and the second assessed fNIRS-derived hemodynamic responses. The MANOVA to assess balance included Path Length, Sway Range, and Sway Velocity as the dependent variables and Condition (TWI vs CWI), Sex (Male vs Female) and Time (Pre-Exposure vs Post-Exposure) as the fixed factors. The MANOVA to assess fNIRS-derived hemodynamic responses utilized O_2_Hb and HHb as the dependent variables and Condition (TWI vs CWI) and Time (Pre-Exposure vs Post-Exposure) as the fixed factors. Prior to conducting analysis, the MANOVA’s normality was verified using Shapiro–Wilks test and sphericity assessed using Mauchly’s Test of Sphericity. If sphericity was violated, a Greenhouse–Geisser correction was applied. For all pair-wise comparisons, a Bonferroni correction was utilized $\frac{k(k-1)}{2}$. The adjusted *p* values were reported. An alpha of *p* ≤ 0.05 was considered significant for all statistical analyses. For all interactions and main effects of each MANOVA, an effect size was computed using partial eta-squared $$\left(\eta \begin{array}{c}{2}\\ {\mathrm{p}}\end{array}\right)$$ calculated as $$\left(\eta \begin{array}{c}{2}\\ {\mathrm{p}}\end{array}\right)= \frac{{\text{Sum of Squares}}_{\mathrm{effect}}}{{\text{Sum of Squares}}_{\mathrm{effect}} + {\text{Sum of Squres }}_{\mathrm{error}}}$$ where 0.01 represents a small effect, 0.06 a medium effect, and 0.14 a large effect (Cohen 1988). All analyses were performed using SPSS statistical analysis software (v. 29, IBM, Armonk, NY, United States).

## Results

**Fig. 2:**
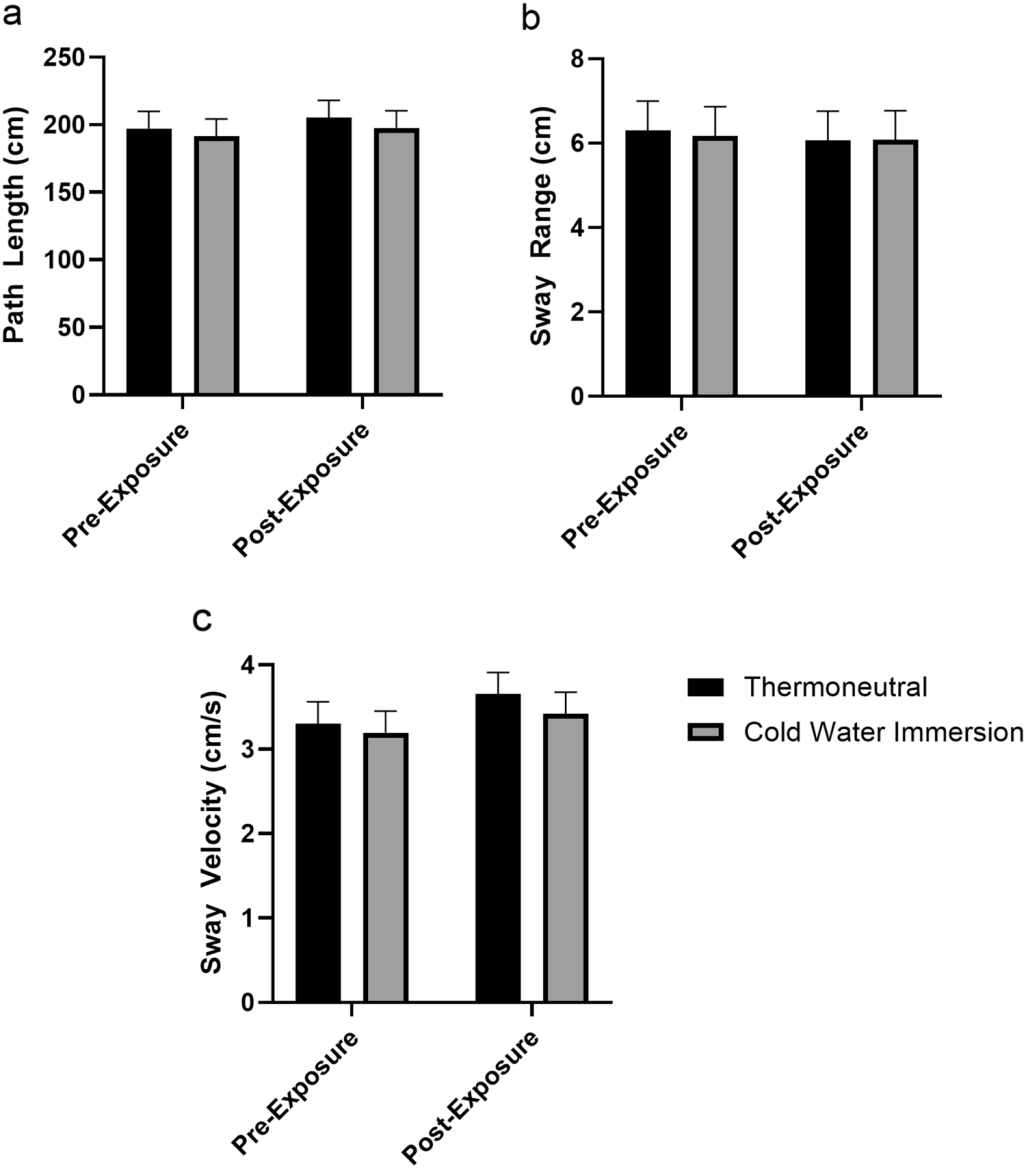
The mean ± SD path length (cm) (**a**), sway range (cm) (**b**), and sway velocity (cm/s) (**c**) results of the 30 s eyes-closed balance assessment pre-exposure and post-exposure to either 10 min of thermoneutral water immersion (35 °C) or cold water immersion (15 °C)

For Path Length (*F*: 1.110; *p* = 0.297; *η* $$\begin{array}{c}{2}\\ {\mathrm{p}}\end{array}$$ = 0.023), Sway Range (*F*: 2.120; *p* = 0.152; *η* $$\begin{array}{c}{2}\\ {\mathrm{p}}\end{array}$$ = 0.042), and Sway Velocity (*F*: 2.302; *p* = 0.090; *η* $$\begin{array}{c}{2}\\ {\mathrm{p}}\end{array}$$ = 0.126), there was no significant difference between Sex; therefore, all statistics for the balance assessment were collapsed across Sex. For Path Length, Sway Range, and Sway Velocity, there were no significant interactions or main effects for Condition, or Time (Table 2; Fig. 2a Path Length, 2b Sway Range, 2c Sway Velocity).

**Table 2:** Statistical results for the condition (CWI vs TWI) × time (pre-exposure vs post-exposure) of path length, sway range, and sway velocity calculated from the 30-s eyes-closed balance assessment

	F statistic	*p* value	Partial eta-squared
Interaction			
Path length	0.006	0.937	0.009
Sway range	0.011	0.917	0.001
Sway velocity	0.060	0.807	0.012
Main effect for condition			
Path length	0.278	0.599	0.003
Sway range	0.008	0.931	0.031
Sway velocity	0.441	0.508	0.009
Main effect for time			
Path length	0.306	0.582	0.013
Sway range	0.059	0.808	0.001
Sway velocity	1.225	0.271	0.010

**Fig. 3:**
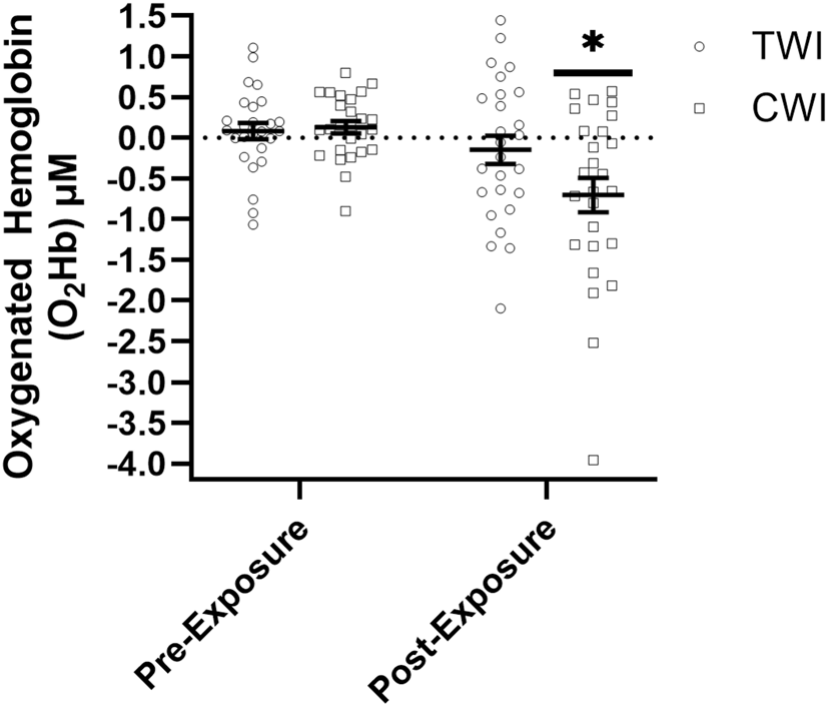
The mean ± SEM for oxygenated hemoglobin concentrations in micromolar (μM) from the prefrontal cortex pre-exposure and post-exposure to either 10 min of thermoneutral water immersion (TWI; ⏺; 35 °C) or cold water immersion (CWI; ⏹; 15 °C) (Mean ± SD). * Indicates Significantly Less (*p* < 0.05) than CWI Pre-Exposure and TWI Pre- and Post-Exposure

For O_2_Hb, there was no significant difference between Sex (*F*: 0.357; *p* = 0.551; *η* $$\begin{array}{c}{2}\\ {\mathrm{p}}\end{array}$$ = 0.004); therefore, all statistics for O_2_Hb were collapsed across Sex. There was a significant Condition × Time interaction (*F*: 8.534; *p* = 0.004; *η* $$\begin{array}{c}{2}\\ {\mathrm{p}}\end{array}$$ = 0.038) for O_2_Hb. Follow-up analyses for the TWI condition indicated no significant differences from TWI Pre-Exposure to Post-Exposure O_2_Hb during the balance assessment (*F*: 2.160; *p* = 0.145; *η* $$\begin{array}{c}{2}\\ {\mathrm{p}}\end{array}$$ = 0.037). The follow-up analyses for the CWI condition indicated a significant decrease in O_2_Hb from Pre-Exposure to Post-Exposure during the CWI condition (*F*: 4.021; *p* = 0.010; *η* $$\begin{array}{c}{2}\\ {\mathrm{p}}\end{array}$$ = 0.108). There were no differences in O_2_Hb between the TWI and CWI conditions at either the Pre-Exposure (*p* = 0.833) or Post-Exposure (*p* = 0.065) timepoints (Fig. 3). Post hoc power analysis for the O_2_Hb model indicated an observed power of 0.826.

**Fig. 4:**
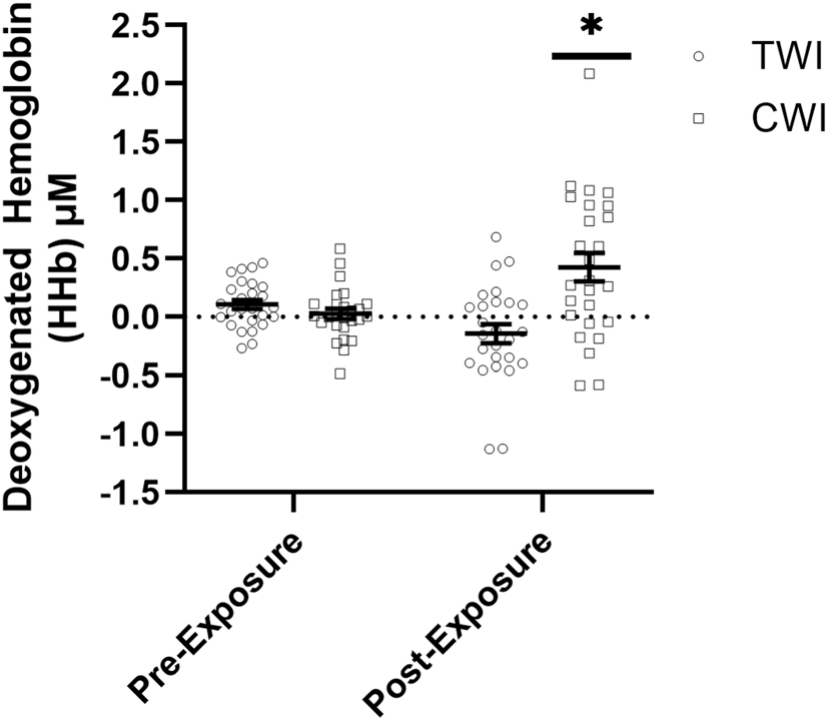
The mean ± SEM for deoxygenated hemoglobin concentrations in micromolar (μM) from the prefrontal cortex pre-exposure and post-exposure to either 10 min of thermoneutral water immersion (TWI; ⏺; 35 °C) or cold water immersion (CWI; ⏹; 15 °C) (Mean ± SD). * Indicates significantly greater (*p* < 0.05) than CWI Pre-Exposure and TWI Pre- and Post-Exposure

For HHb, there was no significant difference between Sex (*F*: 0.082; *p* = 0.775; *η* $$\begin{array}{c}{2}\\ {\mathrm{p}}\end{array}$$ = 0.001); therefore, all statistics for HHb were collapsed across Sex. There was a significant interaction (*F*: 3.978; *p* = 0.038; *η* $$\begin{array}{c}{2}\\ {\mathrm{p}}\end{array}$$ = 0.038) for HHb which indicated no differences from Pre-Exposure to Post-Exposure HHb during the balance task for the TWI condition (F: 1.162, *p* = 0.284; *η* $$\begin{array}{c}{2}\\ {\mathrm{p}}\end{array}$$ = 0.011), but there was an increase in HHb from Pre-Exposure to Post-Exposure during the CWI condition (*F*: 6.290, *p* = 0.002, *η* $$\begin{array}{c}{2}\\ {\mathrm{p}}\end{array}$$ = 0.088). There were no differences in HHb between the TWI and CWI conditions (*p* = 0.932) at Pre-Exposure, but there was a significant difference between conditions at Post-Exposure (*p* = 0.005) (Fig. 4). Post hoc power analysis for the HHb model indicated an observed power of 0.675.

## Discussion

This study examined PFC activation via fNIRS during an eyes-closed balance assessment to better understand the impact of brain function on the maintenance of balance following CWI. Contrary to our hypothesis, 10 min of CWI at 15 °C did not significantly impact postural stability metrics, but did increase the hemodynamic responses over the PFC required to maintain balance compared to TWI (Fig. 2a–c). Specifically, Path Length, Sway Range, and Sway Velocity showed minimal variations between CWI and TWI conditions (3.5%, 2.7%, and 8.5%), respectively. However, CWI induced significant hemodynamic changes in the PFC, which indicated a cold-induced decrease in O_2_Hb and cold-induced increase in HHb during the balance task. Previous studies examining the impact of CWI on balance have reported mixed findings ranging from improved to impaired balance metrics (Tano et al. 2015; Deshmukh and Lilariya 2019). For example, Deshmukh and Lilariya 2019 reported a decrease in single-leg, eye-closed balance following 20 min of below-knee CWI at 15 °C, which was attributed to decreases in foot and ankle strength. In contrast, Tano et al. 2015 indicated that 15 min of CWI at 5 °C resulted in improved eyes-open, but decreased eyes-closed balance, which was associated with slower motor unit action potential conduction velocities, peripheral vasoconstriction, and reduced proprioception. Unlike previous studies, the findings of the current study did not identify any significant differences in eyes-closed balance performance following 10 min of CWI at 15 °C (Fig. 2a–c). Although altered PFC activation was observed following CWI, the brief 30-s balance task may not have been sufficiently demanding to elicit detectable performance decrements. Future studies employing longer or more challenging postural tasks and greater thermal strain are needed to determine whether these neural changes manifest as balance performance impairments. The differences between the postural stability assessment in the current and previous studies may be due to either the duration of the CWI, the severity of the cold stressor, or how the PFC was dysregulated (Tano et al. 2015; Deshmukh and Lilariya 2019). These previous studies did not perform any neurophysiological measurements over the brain, such as fNIRS over the PFC as in the current study. The findings of the current study suggest that PFC regulation and associated metabolic demand play a critical role in maintaining eyes-closed balance assessment (Figs. 3, 4).

Within the current study, there was an increase in HHb and a decrease in O_2_Hb concentrations over the PFC from Pre-Exposure to Post-Exposure during the CWI condition, which were not observed in the TWI condition (Figs. 3, 4). These findings were in alignment with those of Ellis, 2023, who reported a 21% decrease in O_2_Hb over the PFC at rest after 5–10 min of CWI at 10–15 °C when performing the NIH-Examiner and visual categorization task. Although there were similar trends to Ellis (2023), further investigations are needed into the activation patterns throughout the brain under different tasks, as previous work has reported differing brain activation patterns depending on the task being performed (i.e., balance, memory, cognitive, and visual) (Turesky et al. 2018; Smith et al. 2023; Zhang et al. 2025). In addition, the current findings were in agreement with those of Wu et al. 2024, who reported significantly elevated utilization of O_2_Hb over the primary sensory and posterior parietal cortex during a knee proprioception discrimination assessment immediately following 10 min of CWI at 15 °C. Wang et al. 2016 reported high correlations between proprioception and balance in the lower limb, with proprioception being one of the three key sensory mechanisms for maintaining balance (Wang et al. 2016). It is likely that neural activation, balance, and proprioception are closely linked, with impairments in proprioception negatively impacting balance, causing the brain to have greater activation to interpret and modify novel motor pathways. It has been hypothesized that the changes in O_2_Hb following CWI are likely due to greater metabolic demand associated with increased neural demand from thermal sensation, resulting in increased metabolic demand to maintain proprioception and balance (Wu et al. 2024).

To our knowledge, this is the first study to examine the HHb response following CWI with or without a balance assessment. The addition of HHb provides physiological insights that are not captured by O_2_Hb alone, especially in cold stressor research. Specifically, HHb provides additional depth of understanding of the metabolic demand within the brain during cold exposures by quantifying the oxygenation extraction rate of the cerebral tissue (Chen et al. 2020; Nakamura et al. 2020). The increase in HHb diverged from previous studies on cerebral hemodynamic responses that occur during cold exposures, which typically report increased cerebral O_2_Hb and decreased HHb (Minett et al. 2014; Nakamura et al. 2020) (Figs. 2, 3). The hemodynamic responses during cold exposure in the current study likely differed from previous studies because of the differences in when data collection occurred during the cooling and rewarming processes (Minett et al. 2014; Nakamura et al. 2020). Previous studies have examined the hemodynamic shifts during a single cold stressor, without the inclusion of additional motor or cognitive tasks, such as balance (Minett et al. 2014; Nakamura et al. 2020). Initial cold exposures result in rapid cooling of the periphery with a corresponding 24% increase in peripheral resistance, which triggers a 14% increase in overall cerebral blood flow, not regional activation levels, to maintain the tight thermal range needed for cerebral neural function (Brown et al. 2003; Kjeld et al. 2009). In contrast to this hemodynamic response, we hypothesized that during the rewarming phase following CWI, there would be a reduction in O_2_Hb. However, the addition of a cognitive or motor task would likely increase metabolic demand as reflected by an increase in HHb (Figs. 2, 3). This hypothesis is based on the thermoneutral findings of Nakamura et al. 2020, who reported HHb concentration decreasing due to cerebral blood flow exceeding O_2_Hb consumption, with this pattern reversing during the refractory period when two tasks are performed in rapid succession. The hemodynamic responses during the refractory period were attributed to the second task producing a markedly reduced O_2_Hb response and insufficient arterial blood supply (Nakamura et al. 2020). As a result, newly produced HHb from the metabolic demand of the neuronal activities was not diluted or removed from the cerebral tissue at the same rate, leading to an increase in HHb concentration (Nakamura et al. 2020). Thus, in the current study, we hypothesize that the increase in HHb with a concomitant decrease in O_2_Hb likely reflects a refractory period immediately following CWI. This hypothesized refractory period may reflect competing underlying physiological responses of the body coupled with the metabolic demand to maintain balance during the rewarming process. Therefore, these findings suggest that a further examination of the hemodynamic responses occurring during the rewarming phase following CWI should be conducted to include how recovery time and cold stressor severity impact the cerebral hemodynamic responses.

## Limitation

The current study has key limitations that should be considered when interpreting the findings, and caution should be given to not overgeneralize to a large population or across different severities of cold stressors (i.e., more severe cold stressors below 15 °C). Specifically, the sample size of *n* = 26 is modest and limits the ability to generalize to wider populations, especially when considering the potential impact of age and body composition across studies. In addition, the current study only assessed a short, eyes-closed balance assessment; however, future studies should aim to include additional and longer balance and motor tasks while maintaining cold stress. The inclusion of greater physiological data regarding thermal strain also limits the generalization of the study’s findings, and future research should include measures such as core temperature pills and heart rate variability. The current study aimed to limit the impact of diurnal variations in thermoregulation within 1.2 h, which may impact physiological responses and outcome variables within this study; however, a tighter tolerance of time for each visit may improve sensitivity and reduce potential subject-to-subject variations in data.

## Conclusion

In conclusion, this study provides insights into the complex relationship between CWI and postural stability control mechanisms. During CWI at 15 °C, Path Length, Sway Range, and Sway Velocity were not impacted; however, there were differing hemodynamic responses over the PFC to maintain balance. This study contributes to the field by being the first to examine HHb responses following CWI during balance tasks, highlighting an important interplay between recovery from cold stressors, cerebral hemodynamics, and motor control. The observed increase in HHb, coupled with the decrease in O_2_Hb concentrations, suggests a hypothesized refractory period immediately Post-Exposure during the CWI but not the TWI condition. The combined demands of body rewarming and balance maintenance create distinct cerebral hemodynamic patterns. These findings contribute to understanding the cerebral response to cold stress during balance tasks.

## Data Availability

The anonymized data sets are available by request to the corresponding author and approval from Baylor University.
